# Relationship of health-related social needs, social determinants of health, and catheter ablation utilization in atrial fibrillation: Social needs and application of atrial fibrillation ablation

**DOI:** 10.1007/s10840-023-01646-1

**Published:** 2023-09-16

**Authors:** Eva M. Holland, Danielle B. Dilsaver, Ryan W. Walters, Michael H. Kim

**Affiliations:** 1 Dept of Medicine Creighton University School of Medicine, 7710 Mercy Rd, Suite 301, Omaha, NE 68124 USA; 2 CHI Health, Omaha, NE USA

Hospital readmissions after an initial atrial fibrillation (AF) hospitalization represent one of the largest contributors to increased healthcare costs [1]. Catheter ablation of AF has increased over the past two decades and is recognized as an integral treatment for selected AF populations [2]. However, the application of AF ablation has been differentially applied across the population resulting in disparities in care. Prior data has shown female sex, Black race or Hispanic ethnicity, and underinsured patient populations are less likely to receive AF ablation [3]. Furthermore, social determinants of health (SDOH) are associated with higher risk for cardiovascular disease and death in the United States [4]. SDOH include socioeconomic position, race and ethnicity, social support, culture, access to medical care, and residential environments [4]. Individuals with SDOH are a marginalized patient population that may receive effective treatment options at different rates. The impact of SDOH in those that receive AF ablation on associated clinical outcomes is unknown. Health-Related Social Needs (HRSN) quantify many factors related to SDOH [3]. As such, we used the Nationwide Readmissions Database (NRD) to assess the utilization and outcomes of catheter ablation of AF in the presence or absence of a HRSN as defined by five domains (employment, family, housing, psychosocial, and socioeconomic status) previously used by Bensken et al [5].

Index hospitalizations were abstracted from the 2010-2019 NRD for which patients were at least 18 years old with a primary AF diagnosis; hospitalizations were then stratified by whether the patient had a diagnosis with an HRSN domain (ICD codes available on request). We first evaluated whether utilization of catheter ablation for AF differed between those with or without a HRSN diagnosis using the Rao-Scott chi-square test. For hospitalizations that included catheter ablation for AF, we then evaluated differences between those with or without a HRSN diagnosis for 90-day all-cause and cause-specific readmissions, as well as in-hospital mortality, length of stay, and inflation-adjusted hospital cost at index hospitalization [6]. We estimated logistic regression models for readmission and mortality, and log normal regression for length of stay and hospital cost. Multivariable models were also estimated that controlled for age, biological sex, and comorbidity burden [7]. Cause-specific readmissions were indicated by MS-DRG. All analyses accounted for the NRD sampling design with two-tailed p < .05 used to indicate statistical significance.

From 2010-2019, there were an estimated 3,650,995 hospitalizations for AF in the United States, of which 1.2% (95% CI: 1.2% to 1.3%) included a patient diagnosed with at least one HRSN domain. Psychosocial and housing were the most common HRSN domains at 51.7% and 30.5%, respectively.

Hospitalizations that included a patient with a HRSN diagnosis had lower rates of ablation utilization (HRSN: 1.1%, 95% CI: 0.9% to 1.3% vs. no HRSN: 3.8%, 95% CI: 3.7% to 4.0%; p < .001). A paucity of data exist on the association between HRSN and the utilization of AF ablation, and our results demonstrated a disparity in utilization of AF ablation by HRSN status. Despite studies supporting AF ablation improving AF symptoms and reducing adverse cardiovascular outcomes, the HRSN population does not receive this form of AF therapy at the same rate as the AF population without an HRSN diagnosis [2]. As treatment for AF evolves, researching the outcomes of patients with HRSN becomes necessary to prevent further disparity in care and to support the implementation of proven therapies such as AF ablation to marginalized groups that may benefit.

Table 1A presents descriptive statistics for hospitalizations that included catheter ablation of AF for which those with a HRSN were younger, more likely to be male, and in lower income quartiles. Further, hospitalizations that included a patient with a HRSN were more often unplanned, included a weekend admission, and included patients with a greater comorbidity burden. Table 1B shows outcomes for hospitalizations that included catheter ablation for AF stratified by HRSN status. A HRSN was associated with 41% higher adjusted odds of all-cause 90-day readmission (95% CI: 12% to 78% higher, p =.004). Despite this statistically significant association, there was no statistical difference between those with or without a HRSN in the three most common reasons for readmission including heart failure (16.5% vs. 16.6%, respectively, p = .943), cardiac arrythmia (10.8% vs. 14.9%, respectively, p = .066), and sepsis (3.3% vs. 3.6%, respectively, p = .756). As such, the association between a HRSN and increased odds of all-cause readmission were due to the aggregation of less common reasons for readmission that could not be reported individually per NRD data use agreement as observed counts for those reasons for readmission were less than 11. Further, a HRSN was associated with a 73% longer length of stay (95% CI: 58% to 91% longer, p < .001) and 9% higher hospital cost (95% CI: 4% to 15% higher, p < .001); in-hospital mortality could not be reported due to low outcome counts per the NRD Data Use Agreement. Taken together, our results show adverse clinical outcomes after AF ablation for those with a HRSN diagnosis as a HRSN was associated with higher odds of 90-day readmission, longer lengths of stay, and greater hospital cost. Increased awareness of these outcomes could affect treatment protocols to reduce rates of readmission, improving management of AF ablation in patients, and lowering costs within the healthcare system.

**Table 1:** Health related social needs and catheter ablation of atrial fibrillation

A. Descriptives						
Age	HRSN: No	HRSN: Yes	*p*			
	67 [59, 75]	63 [55, 71]	<.001			
Biological Sex						
Male	61.3	68.4	<.001			
Female	38.7	31.6				
Elective Admission	47.7	19.6	<.001			
Weekend Admission	11.2	19.9	<.001			
Primary Payer						
Medicare	61.6	50.5	<.001			
Medicaid	4.6	22.2				
Private	30.2	17.2				
Other	3.6	10				
Income Quartile						
I	22.8	33.8	<.001			
II	25.3	27.6				
III	25.8	21.1				
IV	26.1	17.5				
Elixhauser Comorbidity Index						
For In-hospital Death	0 [-2,6]	1 [-3, 9]	0.031			
For Readmission	6 [-1, 17]	16 [5, 28]	<.001			
B. Outcomes						
	Unadjusted			Adjusted		
	HRSN: No	HRSN: Yes		Ratio (95% CI)		*p*
Index Hospitalization						
In-patient Mortality (%)	0.9	*		*		*
Length of Stay (days)	2.7	5.3		1.73 (1.58-1.91)		<.001
Cost	$29,808	$34,167		1.09 (1.04-1.15)		<.001
All-cause Readmission						
90-day (%)	21.9	33.1		1.41 (1.12-1.78)		0.004

^a^*HRSN* Health-related Social Need

^b^*CI* Confidence Interval

^c^An * indicates that the percentage and result of the statistical test could not be presented per the NRD Data Use Agreement as the number of unweighted hospitalizations was 10 or less

Finally, this study was limited by administrative data as hospitals are not reimbursed for coding a HRSN; thus, HRSN may have been underreported. Further study is needed to delineate the association between HRSN in patients with AF and rhythm control, AF ablation, and clinical outcomes.
